# ROS responsive resveratrol delivery from LDLR peptide conjugated PLA-coated mesoporous silica nanoparticles across the blood–brain barrier

**DOI:** 10.1186/s12951-018-0340-7

**Published:** 2018-02-13

**Authors:** Yang Shen, Bin Cao, Noah R. Snyder, Kevin M. Woeppel, James R. Eles, Xinyan Tracy Cui

**Affiliations:** 1 Department of Bioengineering, University of Pittsburgh, 5057 Biomedical Science Tower 3, 3501 Fifth Avenue, Pittsburgh, PA 15260 USA; 2 Institute of Biomedical Engineering, West China School of Basic Medical Sciences & Forensic Medicine, Sichuan University, Chengdu, 610041 China; 3 Center for the Neural Basis of Cognition, University of Pittsburgh, Pittsburgh, PA 15260 USA; 4 McGowan Institute for Regenerative Medicine, University of Pittsburgh, Pittsburgh, PA 15260 USA

**Keywords:** Blood–brain barrier (BBB), Resveratrol (RSV), Mesoporous silica nanoparticles (MSNPs), Reactive oxygen species (ROS), LDLR ligand peptide

## Abstract

**Background:**

Oxidative stress acts as a trigger in the course of neurodegenerative diseases and neural injuries. An antioxidant-based therapy can be effective to ameliorate the deleterious effects of oxidative stress. Resveratrol (RSV) has been shown to be effective at removing excess reactive oxygen species (ROS) or reactive nitrogen species generation in the central nervous system (CNS), but the delivery of RSV into the brain through systemic administration is inefficient. Here, we have developed a RSV delivery vehicle based on polylactic acid (PLA)-coated mesoporous silica nanoparticles (MSNPs), conjugated with a ligand peptide of low-density lipoprotein receptor (LDLR) to enhance their transcytosis across the blood–brain barrier (BBB).

**Results:**

Resveratrol was loaded into MSNPs (average diameter 200 nm, pore size 4 nm) at 16 μg/mg (w/w). As a gatekeeper, the PLA coating prevented the RSV burst release, while ROS was shown to trigger the drug release by accelerating PLA degradation. An in vitro BBB model with a co-culture of rat brain microvascular endothelial cells (RBECs) and microglia cells using Transwell chambers was established to assess the RSV delivery across BBB. The conjugation of LDLR ligand peptides markedly enhanced the migration of MSNPs across the RBECs monolayer. RSV could be released and effectively reduce the activation of the microglia cells stimulated by phorbol-myristate-acetate or lipopolysaccharide.

**Conclusions:**

These ROS responsive LDLR peptides conjugated PLA-coated MSNPs have great potential for oxidative stress therapy in CNS.

**Electronic supplementary material:**

The online version of this article (10.1186/s12951-018-0340-7) contains supplementary material, which is available to authorized users.

## Background

Oxidative stress induced by excess reactive oxygen and nitrogen species (RONS) can cause inflammation and neuronal death, and has been implicated in various neurodegenerative diseases and injuries [1]. Microglia are a major sources of RONS, and play a crucial role in various brain pathologies [2]. Activated microglia release superoxide (•O_2_^−^) and nitric oxide (•NO) upon activation of NADPH oxidase and nitric oxide synthase (NOS) respectively, both of which can induce neuronal death [3]. To combat RONS, antioxidant compounds have received attention for their prophylactic and therapeutic potentials [4]. Resveratrol (3, 5, 4′-trihydroxy-trans-stilbene, RSV) is well known for its antioxidant, anti-inflammatory, cardio-protective, and neuro-protective properties [5], and has shown its potential in treatment of various disorders mediated by free radicals and oxidative stress [6]. By scavenging RONS and up-regulating the expression of cellular antioxidant and detoxification enzymes including catalase and quinone reductase, RSV improves cellular resistance to oxidative stress.

Unfortunately, RSV has low solubility and the bioavailability is less than 1% for upon systemic administration due to its rapid metabolization in the liver and intestine [7]. Access to the central nervous system (CNS) is further limited by the blood–brain barrier (BBB). The BBB is formed by tight junctions between brain endothelial cells, which protects the CNS from variations in blood composition and toxins and consequently restricts the penetration of various drugs [8, 9].

Nanoparticles (NPs)-based therapeutics have shown great promise due to the flexibility in designing and modifying their compositions and structures for enhanced bioavailability and delivery efficiency, as well as site specific targeting. Over the past few years, significant breakthroughs have been made in the development of various NPs for drug delivery across the BBB [10, 11]. Among these, mesoporous silica nanoparticles (MSNPs) are highly attractive for drug delivery due to their well-defined and controllable microstructure and excellent biocompatibility. With the exceptionally large surface area and pore volume, MSNPs provide greater capacity for drug loading and surface functionalization [12]. Further, MSNPs can be coated with a biodegradable polymer as a gatekeeping layer, enabling controlled drug release. For example, PLA coatings significantly delayed the release of the venlafaxine from mesoporous silica nanosphere under intestinal conditions [13]. Interestingly, ROS may accelerate the degradation of aliphatic polyesters such as PLA [14]. Therefore, we propose to use PLA as a ROS-responsive gate keeper for MSNPs to achieve enhanced drug release under high oxidative stress.

Both degradable polymer and MSNP based nanoparticles have been shown to cross the BBB via adsorptive transcytosis [15]. To enhance the BBB uptake, various efforts have been made to initiate receptor-mediated endocytosis. Specific endothelial receptors, including the transferrin receptor, insulin receptor, leptin receptor, low-density lipoprotein receptors (LDLR), and the low-density lipoprotein receptor-related proteins are known to be localized on the brain vascular endothelial cells and mediate transcytosis across the BBB via ligand–receptor binding. Attaching ligands of these receptors to NPs may significantly increase their transcytosis and result in higher delivery into the CNS tissue. Recently, Malcor et al. [16] identified a new peptide (Ac-[cMPRLRGC]c-NH_2_) as a ligand for LDLR. This peptide has shown the ability to cross spinal-blood barrier with high efficiency and speed, suggesting its potential as vectors for CNS targeting. To our knowledge, this peptide has not been utilized to enhance the nanoparticle delivery across BBB.

**Fig. 1:**
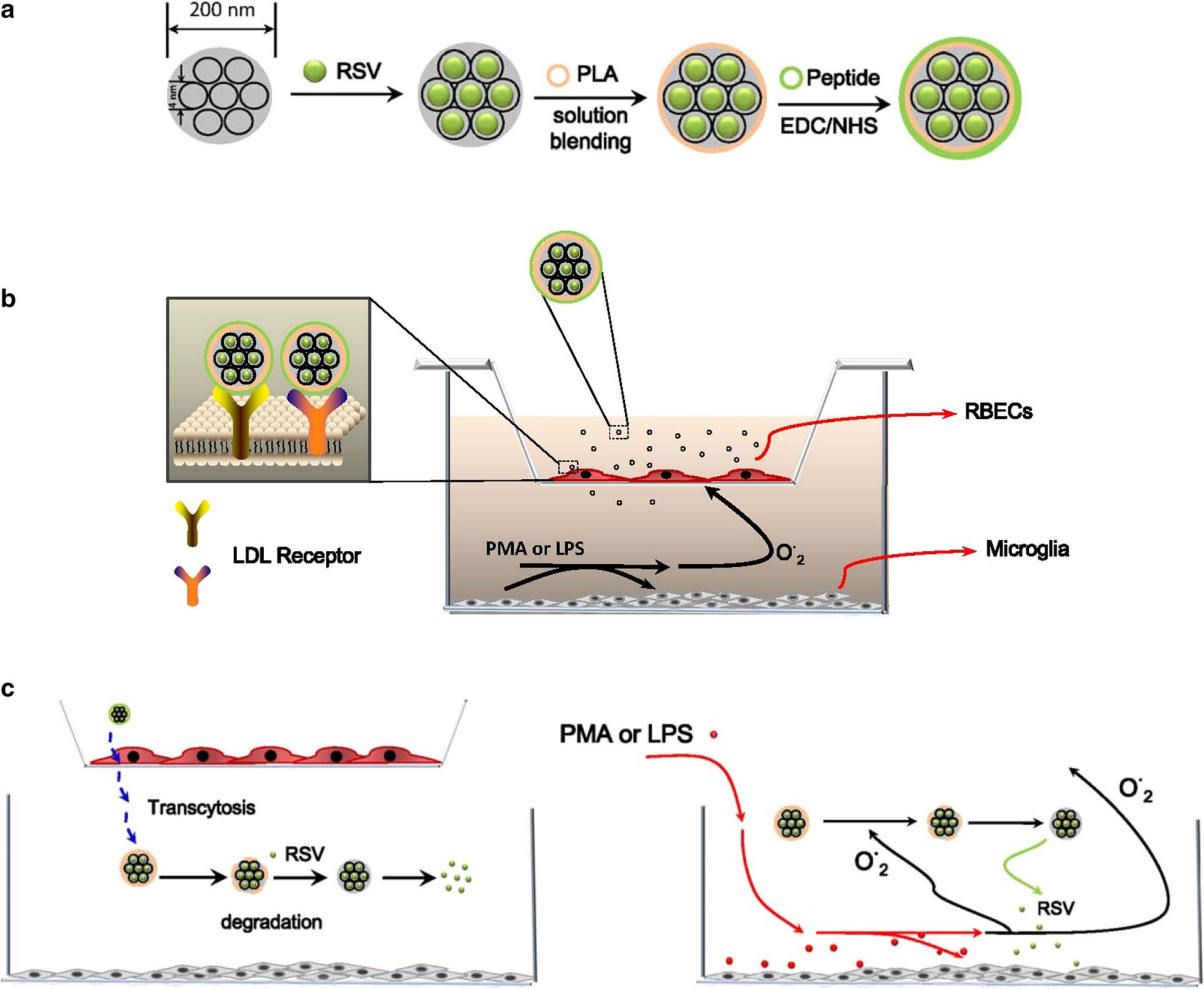
Schematic of MSNPs delivery system. **a** Design of RSV loaded MSNPs with peptide bind PLA-coating. **b** An in vitro BBB model with RBECs cultured on semi-permeable membranes of Transwell chamber with the microglia cells grown on the bottom of the well. MSNPs with covalently attached LDL ligands are recognized and internalized by LDL receptors of RBECs. PMA or LPS stimulations activate the inflammatory response of microglia to release reactive species. **c** PMA or LPS stimulated microglia produce superoxide or nitric oxide which can accelerate the degradation of PLA and accelerate RSV release to control the oxidative stress

In this study we report the development of a PLA coated and LDLR ligand peptide functionalized MSNP delivery system that can actively cross BBB via receptor-mediated transcytosis, and release antioxidant RSV upon ROS stimulation with the goal of treating neurological diseases caused by oxidative stress. The schematic of the design is shown in Fig. 1. MSNPs were loaded with RSV and encapsulated by PLA as the gatekeeper. Next, the LDL peptide ligand was covalently attached to the PLA layer via EDC/NHS chemistry (Fig. 1a). The receptor-mediated transcytosis and ROS stimulated RSV release was demonstrated by an in vitro BBB model, composed of RBECs and microglia HAPI (highly aggressively proliferating immortalized) cells co-cultured in a Transwell (Fig. 1b). We show that the RBECs on the top well can form tight junctions and create a transport barrier mimicking the BBB, while microglia on the bottom wells could be stimulated with phorbol-myristate-acetate (PMA) or lipopolysaccharide (LPS) to produce abundant superoxide or nitric oxide. We have demonstrated that the addition of LDL peptides to the particle surface could improve the transcytosis of MSNPs across BBB, and once MSNPs arrive at the microglia side, the PLA coating was degraded by the high concentration of RONS produced by microglia and RSV were released subsequently to reduce inflammation (Fig. 1c).

## Methods

### Materials and reagents

Mesoporous silica nanoparticles (particle size 200 nm, pore size 4 nm), Poly(l-Lactide) (Mn 5000), *N*-hydroxysuccinimide (NHS, 98%) and *N*-(3-dimethylaminopropyl)-*N*′-ethylcarbo-diimide (EDC, > 97%), Xanthine oxidase (XO, ≥ 0.4 units/mg protein) and 2-amino-4-hydroxypteridine Pterine (PR) were purchased from Sigma (St. Louis, MO). RSV was purchased from Calbiochem^®^ (EMD Millipore, USA). The FITC-labeled LDL peptide ligand was synthesized by Bankpeptide Inc (Hefei, China) according to the previous report [16]. The sequence of the peptide is Acp-(Cys-Met-Pro-Arg-Leu-Arg-Gly-Cys)c-NH_2_. The N-terminal of peptide was labeled by green fluorescent FTIC, and the C-terminal was amidated.

The primary antibodies, anti-Vinculin and anti-Iba1 mouse monoclonal antibody, anti-Myosin, anti-iNOS, and anti-NADPH p47^phox^ rabbit polyclonal antibody were purchased from EMD Millipore (Billerica, MA, USA). The anti-occludin rabbit polyclonal antibody was purchased from Lifespan Biosciences, Inc. (Seattle, WA, USA).

### The drug loading on the MSNPs

Resveratrol was dissolved in 50% ethanol/PBS solution. The absorbance of different concentrations of RSV was examined by a Gemini XPS microplate reader (SpectraMax i3x, Molecular Device, UK) at wavelength 304 nm, and the standard curve was determined. The 10 mg MSNPs were dispersed in 50% ethanol/PBS solution with 30 μg/mL RSV at room temperature. The mixed solution was sonicated and stirred to allow MSNPs passively adsorb drugs. Ever 4 h at first day and every 24 h at a few days later, the mixture was centrifuged for 10 min at 3000×*g*, and the supernatant was analyzed with spectroscopy at 304 nm to determine the amount of RSV remaining in solution. The amount of drug loaded was calculated by subtracting the amount of free RSV. This drugs adsorption was stopped until the amount of RSV loading was no longer increased markedly. Three specimens for each treatment condition were repeated for two times.

### PLA coating and peptide binding on the MSNPs

The solution blending method was used to coat the MSNPs with PLA layer as the gatekeeper for controlling release of RSV [17]. Briefly, 10 mg of RSV-loading MSNPs were dispersed in a solution containing 10, 5, or 2.5 mg PLA (mass ratio 1:1, 1:0.5 and 1:0.25) in 10 mL of chloroform, respectively. After sonication and stirring for 30 min, the PLA-coated particles were centrifuged for 10 min at 3000×*g* in a glass centrifuge tube. The supernatant was discarded and PLA-coated MSNPs (PMSNPs) were collected. The FITC-labeled LDL peptides were conjugated to the surface of MSNPs using an EDC/NHS technique. Briefly, 10 mg PLA coated-MSNPs was added to 0.2 M EDC and 0.1 M NHS mixture for 1 h stirring to activate the –COOH group of PLA, followed by centrifuge and PBS wash to remove EDC/NHS. Next, activated PLA–MSNPs were resuspended in 10 μg/mL peptide solution for 4 h stirring at room temperature. To determine if the peptides were covalently conjugated or passively absorbed, the PLA-coated MSNPs were mixed with peptides and washed by PBS in absence of EDC/NHS as controls. The modified MSNPs were centrifuged at 3000×*g* for 10 min to remove free peptide. The LDL peptide bound PLA-coated nanoparticles (LPMSNPs) were characterized by handheld ultraviolet lamps (upland, CA, USA). The fluorescence intensities of nanoparticles were then examined by a Gemini XPS microplate reader (SpectraMax i3x, Molecular Device, UK) at 525 nm using a 96-well black polystyrene plate (Corning Costar^®^, USA). The sample identification codes and preparation conditions were described in Additional file 1: Table S1.

### Surface characterization by TEM and SEM

The morphology and pore size of PLA-coated MSNPs were characterized using transmission electron microscopy (TEM, JEM-1400 Plus, Japan) operating at an accelerating voltage of 80 kV. A minimum of five images for each sample were captured and representative images are shown with magnification of 100,000× and 250,000×, respectively. The PLA-coated or uncoated MSNPs were mounted on a stub of metal, coated with 40–60 nm gold, the morphology of all specimens were also imaged by scanning electron microscopy (SEM, JSM-6335F, JEOL, USA).

### NMR

To determine the amount of PLA coated on MSNPs at different initial PLA:MSNP ratio, the MSNPs were examined by nuclear magnetic resonance (NMR, Avance III HD, Bruker, USA). The MSNPs were dried and re-dissolved in 5 mL deuterated chloroform (CDCl_3_) solvent. The samples were then examined using ^1^H NMR at 400 MHz with 32 scans. The concentration of PLA in the sample was calculated based on the integrated peak intensity of ^1^H NMR peaks specific to known concentrations of pure PLA and the final peaks gathered from PLA coated MSNPs.

### In vitro drug release profile

To determine if the superoxide is able to accelerate degradation of PLA coating, the mass reduction of PLA was firstly examined by weighing. Briefly, the 100 mg pure PLA were placed into 10 mL pH 7.4 PBS solution containing abundant superoxide (produced by addition of XO and PR) and hydrogen peroxide (H_2_O_2_). 10 nM XO and 100 µM PR were added to the medium once per hour. The residual PLA mass were accurately weighed after 3 and 5 day and mass reduction were calculated accordingly.

Further, the release of RSV from the PLA-coated MSNPs in PBS solution was evaluated. 10 mg of MSNPs with or without PLA coating were suspended in 10 mL PBS. 10 nM XO and 100 µM PR were similarly added to the medium once per hour. Three 100 μL samples (100 μL per well in a 96-well half area plate) were taken out from the medium at specified time point for measuring RSV concentration using SpectraMax i3x microplate reader. After measurement, the collected samples were placed back to medium. The concentration of RSV released was calculated as a percentage of the total absorbed RSV in MSNPs.

### Isolation and culture of RBECs

All animal work was performed under the guidelines of the University of Pittsburgh Institutional Animal Care and Use Committee (IACUC). The isolation of primary rat brain microvascular endothelial cells was previously well-described [18]. Briefly, 5 week old sprague-dawley rats were euthanized and the cerebral hemispheres were separated and placed in cold HBSS buffer supplemented with penicillin 100 units/mL and streptomycin 100 μg/mL. After removing the meninges from the forebrains, the brain tissues were crushed in Dounce homogenizer and subsequently digested with an enzymatic solution for 1 h at 37 °C. The suspension were then mixed with 25% BSA/HBSS and separated by density dependent centrifugation at 3600*g* for 15 min. The sediment was then digested by mixed enzymatic solution again. The tissue was washed with HBSS and centrifuged at 1000×*g* for 5 min, and the sediment was resuspended in DMEM/F12 supplemented with 20% bovine serum, 2 ng/mL basic fibroblast growth factor, 100 μg/mL heparin, 50 μg/mL gentamicin and 2.5 mM HEPES with 4 μg/mL puromycin. The suspension was transferred to flasks with pre-coated collagen type IV and fibronectin (BD Medical Supplies, USA). The passage 2–5 cells were used in this study.

The brain-derived microglial cell line HAPI cells were kindly provided by Dr. Xiaoming Hu (Department of Neurology, University of Pittsburgh). HAPI cells were passaged at a density of 1 × 10^5^ cells/cm^2^ in 48-well plate. Cells were grown until ~ 80% confluence (24 h) in 10% fetal bovine serum containing media (Invitrogen, Carlsbad CA). Stimulation and treatments described below were carried out in serum-free DMEM media. All cells were washed and bathed in serum-free media the night before oxidative species measurements.

### Analysis of cellular uptake

The cellular uptake and location of NPs were examined using TEM. The RBECs (between passage 2–5) were cultured until 90% confluence, and then 0.5 mg/mL peptide conjugated PLA-coated MSNPs was added to cell media for 24 h and then gently collected. The collected samples were centrifuged at 1200 rpm, fixed with 0.5% glutaraldehyde, and 3% glutaraldehyde was slowly added. All samples were fixed by 1% OsO_4_, dehydrated by different concentrations of acetone, embedded by epoxy resin (Epon812), cut into slices and doubled dyed using uranyl acetate and lead citrate. The microstructure of cells and nano-micelles was observed by TEM (JEM-1400 Plus, Japan). Additionally, the RBECs were fixed with 4% paraformaldehyde, and incubated in the DiI cell-labeling solution for 10 min at room temperature. The cellular uptake and location of NPs were also examined using laser scanning confocal microscopy (Leica DMI4000 B, Germany).

### BBB model building and immunofluorescence assay

In vitro BBB model was built with RBECs and HAPI cells co-culture through Transwell porous polycarbonate membrane (0.4 μm pore size) in a 24-well carrier plate (Thermo Scientific, USA). In the RBECs/microglia HAPI cells co-culture system, the HAPI cells at a density of 1 × 10^4^ cells/cm^2^ were first cultured in the 24-well carrier plate, the RBECs at a density of 5 × 10^4^ cells/cm^2^ were cultured in the inner wells of Transwell chamber coated with collagen IV and fibronectin (BD Medical Supplies, USA). The same density of RBECs and HAPI cells were also simultaneously cultured in another 24-well plate to observe the cell growth. The paracellular permeability was monitored using measurement of transendothelial electrical resistance (TEER). For the measurement of TEER, two electrodes were separated by the endothelial layer, i.e., one electrode was placed in the inner well and the other in the lower chamber. BBB monolayers with TEER above 200 Ω cm^2^ were used for the nanoparticle transport studies. The RBECs and HAPI cells cultured in another 24-well plate were stained using double immunofluorescence. Cells were fixed with 4% paraformaldehyde, then blocked and permeabilized with 5% goat serum and 0.2% Triton X-100 in PBS for 30 min. Following blocking and permeabilization, samples were incubated in the primary antibody (anti-Occluding, anti-Vinculin, anti-Myosin diluted in PBS with 5% goat serum, with 1:100 dilution) at 4 °C overnight, respectively. After washing with PBS for three times, the secondary FTIC-conjugated and PE-conjugated immunoglobulin diluted in PBS with 5% goat serum (1:1000 dilution) were mixed and incubated at room temperature for 45 min to conjugate primary antibody. The DAPI with 1:800 dilution was added for nuclei staining for 10 min. Samples were observed by fluorescence microscope.

### Superoxide and nitric oxide production assay

The HAPI cells produce superoxide and nitric oxide under the condition of PMA or LPS stimulation, which were analyzed by cytochrome C reduction and nitrite production, respectively. Prior to Transwell model, the effect of RSV on HAPI cells stimulated by PMA and LPS were examined. The HAPI cells with 90% confluence in 24-well plate were pre-incubated in presence of different concentration of RSV (1, 5, 10 μg/mL) for 3 h. 10 ng/mL LPS (derived from gram-negative bacteria-derived *Escherichia coli*) and 2 ng/mL interferon gamma (IFNɣ) were subsequently added into culture medium at 37 °C for 24 h. Using the commercially available Griess reagent kit for nitrite determination (Molecular Probes, Inc., USA), the nitric oxide production in supernatant was measured by absorbance at 540 nm. Superoxide production by PMA-activated HAPI cells is measured with the cytochrome C, which is reduced by superoxide into detectable ferrocytochrome c. The HAPI cells were pre-incubated in presence of different concentration of RSV for 3 h, and then 10 ng/mL PMA was added. After 24 h, 100 μg/mL cytochrome C was added into medium and continued to culture for 3 h. The ferrocytochrome c is measured at 550 nm using a 96-well UV half area plate (Corning, USA) and the reduction of cytochrome C was determined.

### LPMSNPs across the BBB

In vitro BBB model co-cultured with RBECs and HAPI cells was described in “BBB model building and immunofluorescence assay”. Prior to PMA or LPS/IFNɣ stimulation, the 1 mg/mL LPMSNPs were firstly co-cultured with RBECs at inner wells of Transwell chamber for 24 h in the presence of serum. The 10 ng/mL PMA or 10 ng/mL LPS/2 ng/mL IFNɣ was then added to activate HAPI cells. The absorbance of RSV from lower culture medium in 24-well plate was measured at 304 nm. At the same time, the supernatant was taken out from the inside of Transwell chamber, and measured at 525 nm using a 96-well black polystyrene plate (Corning Costar^®^, USA) to determine residual FITC-labeled LPMSNPs. The superoxide/nitric oxide production assay, as described in “Superoxide and nitric oxide production assay”.

The effects of released RSV on expression of NADPH oxidase marker p47^phox^, Iba-1 and iNOS were further examined by quantitative image analysis. The sample preparation is the same as described in “BBB model building and immunofluorescence assay”. Samples were imaged by laser scanning confocal microscopy (Leica DMI4000 B, Germany), with constant setting in all the samples. Image processing and data quantification of the area and of the intensity of fluorescence images were performed with the software ImageJ 1.48v (National Institutes of Health, Bethesda, MD, USA). At least five randomly chosen fields for a total of minimum 30 cells were measured in each sample. Fluorescence intensity is given in arbitrary units as an average value per cell in the selected representative fields.

### Statistical analysis

The data were analyzed using statistical software SPSS 11.5 (SPSS, Inc., Chicago, Illinois). Data obtained from different treatment groups were statistically compared and reported as mean ± standard deviation. To reveal differences among the groups, one-way ANOVA followed by Tukey’s test was used. Differences were considered significant at *p* < 0.05.

## Results

### Characterization of PLA-coated MSNPs (PMSNPs)

**Fig. 2:**
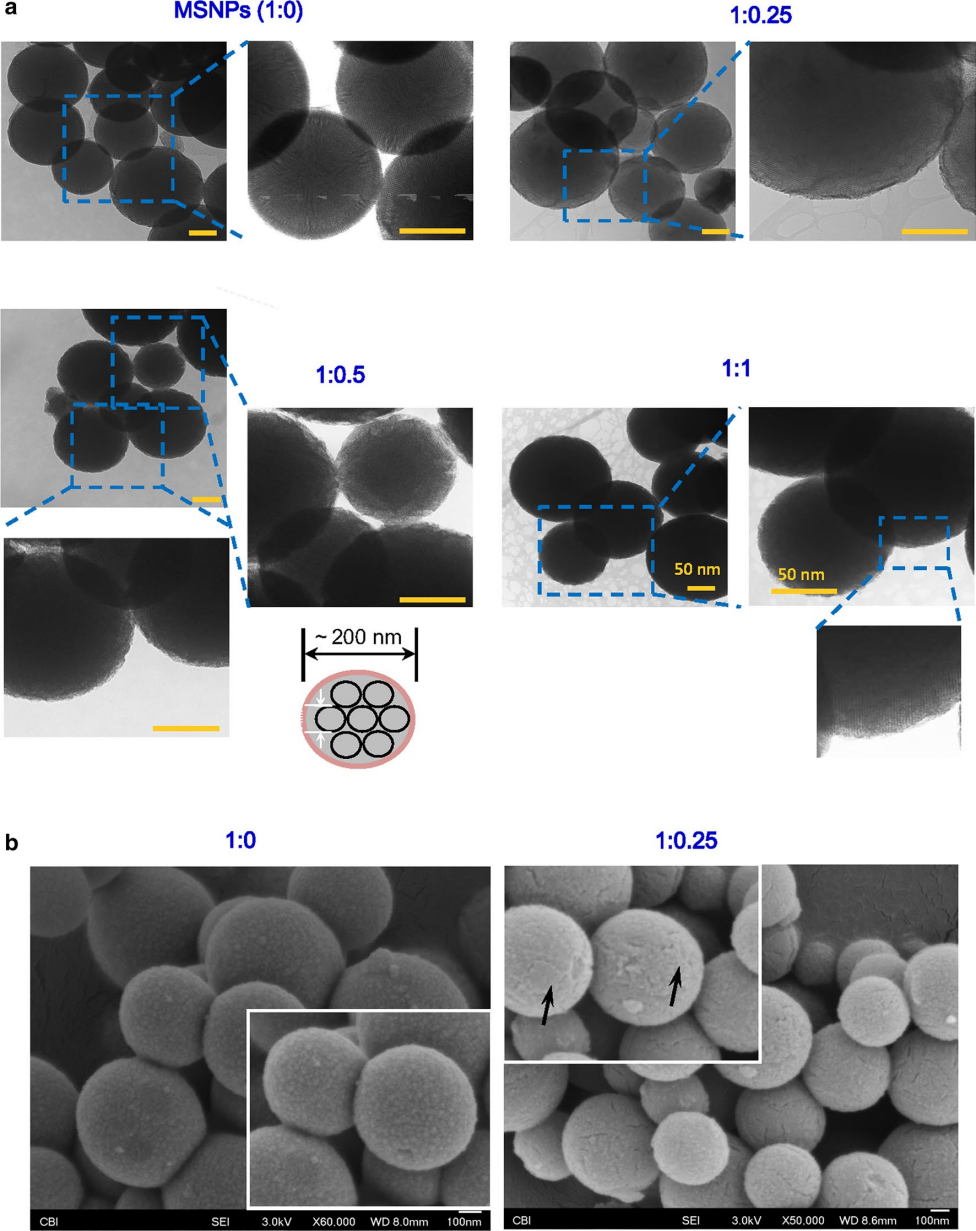
Morphological characterization of PLA-coated MSNPs. **a** TEM images of PLA-coated MSNPs with different mass ratio (1:0, 1:0.25, 1:0.5 and 1:1). Scale bar = 50 nm. **b** SEM images indicate the difference of surface morphology of MSNPs with (1:0.25) or without (1:0) PLA coating

The commercial MSNPs with 200 nm particle size and 4 nm pore size were coated by PLA with different mass ratios and characterized by TEM (Fig. 2a). The enlarged image clearly shows the mesoporous structure and smooth border in uncoated MSNPs with arrays of nanochannels open at the surface (1:0 group). On the other hand, the TEM images of MSNPs coated with PLA polymer in 1:0.25, 1:0.5 and 1:1 group still show visible arrays of one-dimensional channels in the interior, but with a thin amorphous layer of material at the outmost surface. These images indicate that the PLA coating did not fill the interior pores, but simply covering them at the exterior. With increasing concentration of PLA, the borders of MSNPs become obscure. To further verify the PLA coating, the surface of samples were examined by SEM. As shown in the SEM images, the surface of MSNPs without PLA coating (1:0) is uniform, while cracks (indicated by black arrows in Fig. 2b) are found on the surface of PLA-coated samples (only 1:0.25 group is shown here), presumably due to the cracking of the PLA layer upon drying (Fig. 2b). TEM and SEM results provide evidence that PLA polymer, as a gatekeeper, can be coated on the MSNPs by solution blending method. We eluted the PLA coating from MSNPs and subsequently characterized the peak area of PLA using NMR (Additional file 1: Figure S1). The result shows that the ratio of PLA peak among 1:0.25, 1:0.5 and 1:1 groups are approximately equal to 1:2:4 (4.06:7.85:15.68), indicating that increasing amount of PLA coating are present on the MSMPs when increasing the mass ratios of PLA/MSNP in the initial coating solutions (Additional file 1: Figure S1).

### In vitro RSV adsorption and release

The quantitative analysis of RSV loading and release was determined by absorbance of RSV at 304 nm. The highest loading content of RSV into MSNPs is ~ 16 μg/mg (RSV:MSNPs, w/w) after 3 days of loading (Additional file 1: Figure S2).

**Fig. 3:**
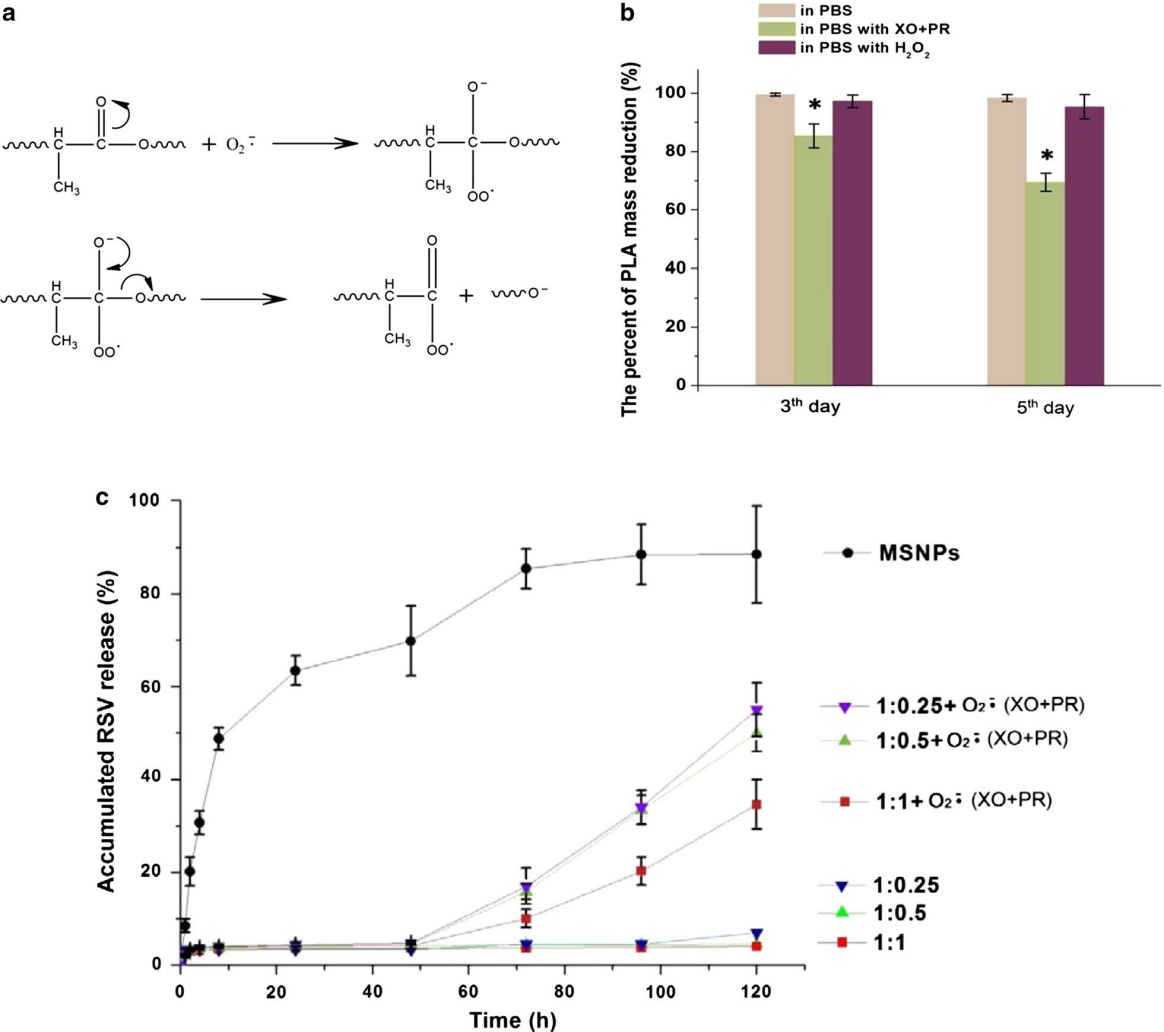
RSV release in vitro. **a** Scheme of superoxide ion accelerating the hydrolysis of PLA by breaking the aliphatic ester bonds (modification based on Ref. [14]). **b** Superoxide generated by XO and PR accelerate the degradation of PLA. *p < 0.05 vs control (PBS only); **c** In vitro release of RSV from uncoated and PLA-coated MSNPs in PBS with and without the presence of superoxide production (XO + PR). n = 3

The release profile of RSV from various PLA/MSNPs was quantified as shown in Fig. 3c. Without the PLA coating, the MSNPs rapidly released RSV in PBS with a burst release of 50% in the first 24 h and reached 90% release after 5 days. With PLA coating, only about 5% RSV was released over the 120 h for all three PLA/MSNP ratios. Clearly, PLA served as a gatekeeping layer and effectively delayed the RSV release from MSNPs. Superoxide has been reported to nucleophilically attack the aliphatic polyester structure in PLA and cause chain fragmentation (proposed mechanism shown in Fig. 3a). To examine the PLA degradation by superoxide, pure PLA polymer was placed in PBS buffer containing OX/PR (producing superoxide) or hydrogen peroxide (H_2_O_2_). In PBS buffer with OX/PR, the mass of PLA samples decreased by 16% at 3 days and 31% at 5 days after exposure, while the samples in H_2_O_2_ did not lose weight (Fig. 3b). This suggests that superoxide is able to accelerate the degradation of PLA. The drug release profile showed that RSV started to release after 48 h, and released from 30% (1:1 group) to 55% (1:0.25) after 5 days in the presence of XO/PR (Fig. 3c), indicating that superoxide can effectively trigger the drug release by accelerating the degradation of the PLA gatekeeper.

### LDL peptide-bound MSNPs (LPMSNPs) and RBECs uptake

**Fig. 4:**
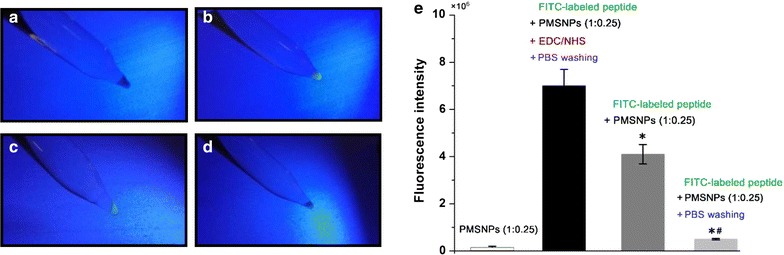
Verification of FITC labeled LDL peptides binding to PLA-coated MSNPs (only 1:0.25 showed) using EDC/NHS method. **a** No fluorescence observed from PMSNPs without the peptide. **b** MSNPs with PLA and fluorescent LDL peptide coating (1:0.25) shows strong fluorescence intensity, which is stable after multiple centrifugation washes. **c** The MSNPs with PLA coating passively adsorb LDL peptides in the absence of EDC/NHS and showed weak fluorescence intensity, and the fluorescent LDL peptides can be washed off by PBS (**d**). **e** Quantification of fluorescence intensity of PMSNPs in the absence or presence of FITC-LDL peptides with or without EDC/NHS. *p < 0.05 vs FITC-peptides + PMSNPs in presence of EDC/NHS; ^#^*p* < 0.05 vs FITC-labeled peptides + PMSNPs in absence of EDC/NHS

The PMSNPs were conjugated with prepared LDL peptides using EDC/NHS chemistry. As seen in Fig. 4, PMSNPs without LDL peptide binding does not fluoresce (Fig. 4a), while intense fluorescence could be found in FITC-LDL peptide bond PLA-coated MSNPs (Fig. 4b). This binding is stable as indicated by the fluorescence intensity after several centrifuging and PBS washes. To determine if the LDL peptides were simply absorbed on PLA-coated nanoparticles instead of covalently bound, the PMSNPs were mixed with the FITC-LDL peptides in absence of EDC/NHS. The PMSNPs turned fluorescent after this treatment (Fig. 4c), but fluorescence was lost after washing and centrifugation with PBS (Fig. 4d). The fluorescence intensities of PMSNPs with different treatments were quantified and compared, (Fig. 4e) and the results were consistent with the qualitative observation. The treatment by FITC-LDL peptide and EDC/NHS cross linker resulted in the highest fluorescence intensity. The treatment with peptide but without EDC/NHS resulted in an intensity 30.1% weaker, which further decreased by 89% after washing (Fig. 4e). Together these results confirmed that LDL peptides ligand could be effectively conjugated with PMSNPs by EDC/NHS method.

**Fig. 5:**
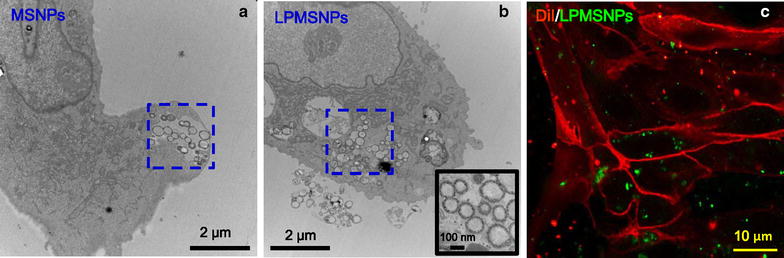
Internalization of LDL peptide decorated PLA-coated MSNPs by RBECs. **a** TEM image of MSNPs (indicated by blue square) internalized by a RBEC. **b** TEM image of LDL peptide decorated PMSNPs (indicated by blue square) internalized by a RBEC, scale bar = 2 μm. The inset is an enlarged TEM image verifying the circular structures in side the cell are indeed mesoporous MSNPs. Scale bar = 100 nm. **c** Fluorescent images showing RBECs (cell membranes were stained with DiI, red fluorescence) cultured with LPMSNPs (green fluorescence). Many green LPMSNPs are seen inside of the RBEC cells. Scale bar = 10 μm

To determine the uptake of the particles by RBECs, the MSNPs and FITC-labeled LDL peptides conjugated with PMSNPs (LPMSNPs) were co-cultured for 24 h with cells and subsequently analyzed by TEM and confocal fluorescence microscopy. As shown in Fig. 5, TEM images provide the strong evidence that MSNPs could be effectively internalized by RBECs. Compared to MSNPs without PLA coating, the RBECs appeared to uptake more LPMSNPs (indicated by blue square in Fig. 5a, b), qualitatively suggesting that the LDL peptides may enhance the internalization and transcytosis of MSNPs, although further studies to better quantify the cell internalization have to be made. TEM results also provide detailed localizations of the intracellular micelles. It is evident that both of MSNPs and LPMSNPs located inside cytoplasm. Additionally, the higher magnification of TEM image in Fig. 5b inset reveals the mesoporous structure of MSNPs inside the cells. Additionally, the green fluorescence in cells was observed (Fig. 5c), indicating that LPMSNPs can be efficiently transported into RBECs and accumulate at its target site.

### Characterization of the in vitro BBB model

**Fig. 6:**
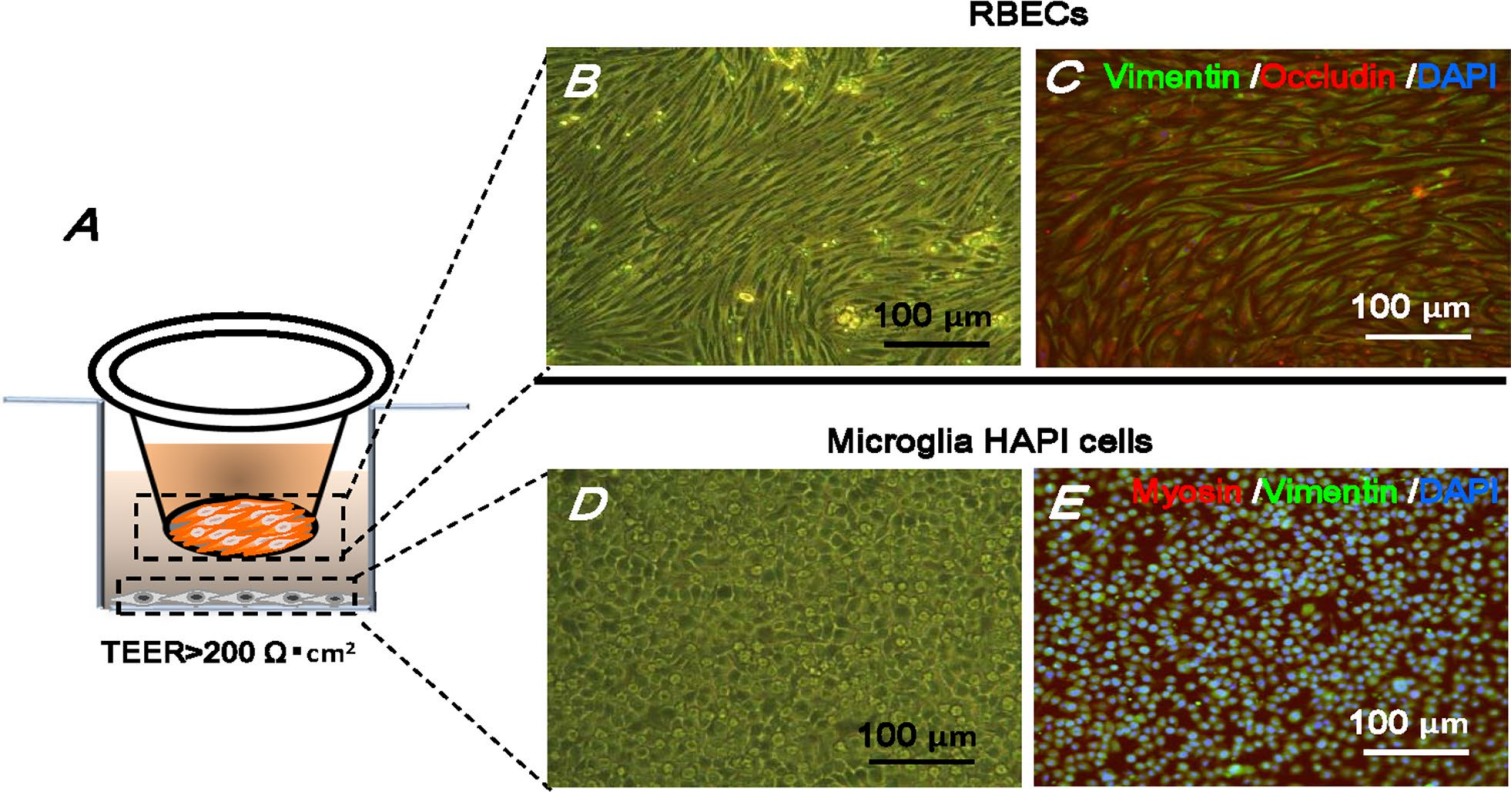
The BBB model. **A** RBECs are cultured on semi-permeable membranes of Transwell chamber with the HAPI microglia cells grown on the bottom of the well. BBB monolayers with TEER above 200 Ω cm^2^ were used for transport studies. The same density of RBECs and HAPI cells are also culture in 24-well plates for immunofluorescent staining and imaging. **B**, **D** RBECs and microglia HAPI cells were visualized under light microscope respectively. **C** RBECs were stained with fluorescently labeled antibodies against Vimentin (green) and occludin (red). **E** HAPI cells were stained with antibodies against Vimentin (green) and Myosin (red). The nucleus is stained by DAPI (blue), scale bar = 100 μm

In vitro BBB model was built with RBECs and microglia HAPI cells co-cultured through a 0.4 μm porous Transwell membrane in a 24-well plate (schemed in Fig. 6A). The RBECs were grown on the membrane of the insert and the HAPI microglia cells were plated on the bottom of the well. To clearly observe the density and morphology of these two types of cells, the RBECs at a density of 5 × 10^4^ cells/cm^2^ and HAPI cells at a density of 1 × 10^4^ cells/cm^2^ were simultaneously cultured in another 24-well plate. After 5 days, both types of cells were grown over 90% confluence, and were marked using double immunofluorescence staining. The actin-binding proteins (Vimentin and Myosin) and tight junction component (Occludin) are used to show the morphology and cell–cell junction of RBECs and HAPI cells, respectively. The images of standard bright-field and immunostaining fluorescence both indicated that RBECs grew in non-overlapping continuous monolayers and exhibited growth inhibition at confluence with typical elongated spindle-shaped morphology (Fig. 6B, C). Figure 6D, E showed the non-activated microglia with round cell bodies and dark nuclei (double immunostaining of Vimentin and Myosin).

### LPMSNPs cross the BBB and release RSV

**Fig. 7:**
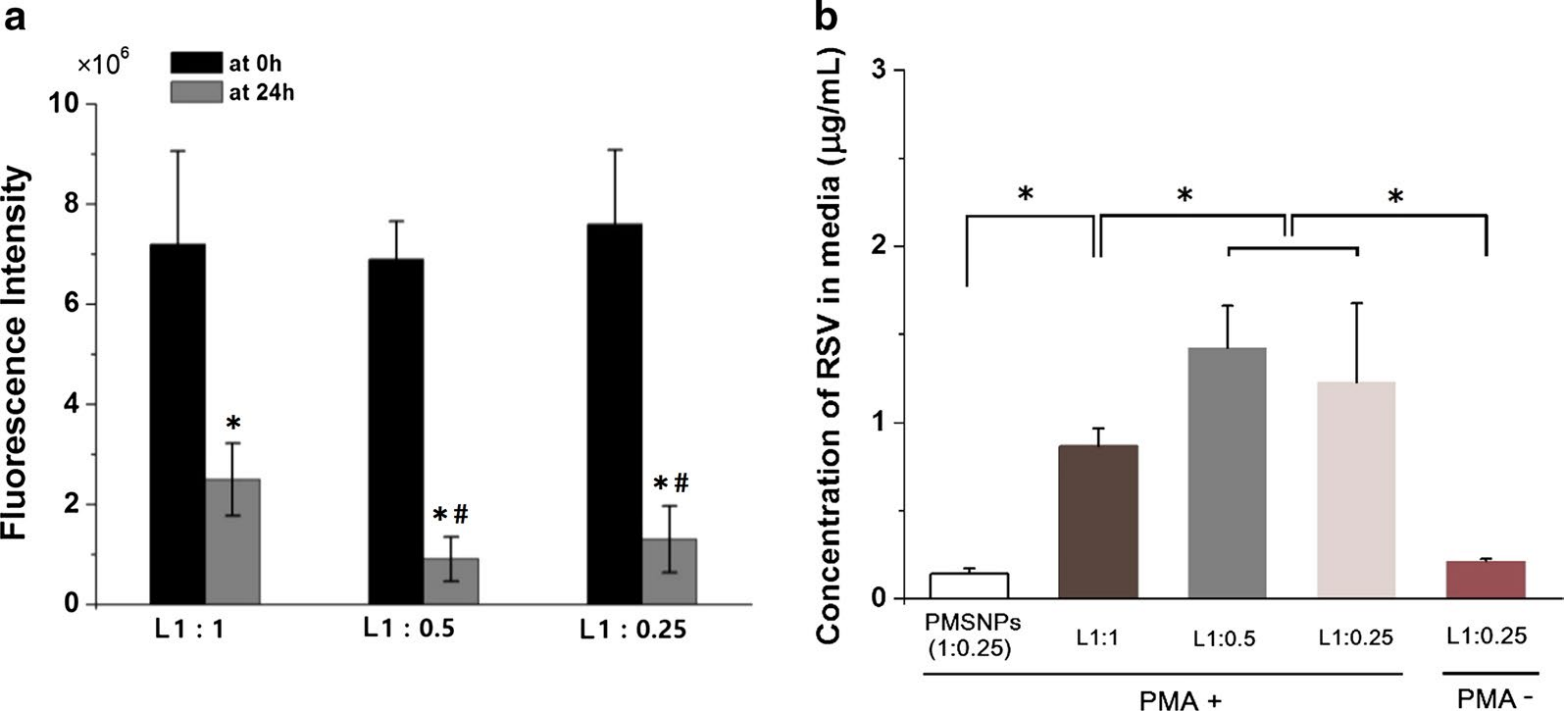
Quantitative analysis of LPMSNPs across the in vitro BBB model. **a** The fluorescence intensity of FTIC-labeled LPMSNPs in upper Transwell chamber at 0 and 24 h. Significant decrease in fluorescence intensity was observed after 24 h suggesting transport of MSNPs across the BBB. **b** The concentration of RSV in lower culture medium released from different NPs groups in presence and absence of PMA stimulation. Higher RSV release was observed in all peptide bound LPMSNPs upon PMA stimulation. The minimum RSV release was observed from PMSNPs without peptide. *p < 0.05 vs 0 h; ^#^*p* < 0.05 vs L1:1 at 24 h

The transport across the BBB of LPMSNPs was analyzed by the Transwell model. The PMSNPs and LPMSNPs were firstly added in Transwell chamber for 24 h before PMA stimulation. As shown in Fig. 7a, the fluorescence intensity of all LPMSNPs (L1:1, L1:0.5 and L1:0.25) sharply decreased in upper chamber at 24 h, which showed significant differences compared with baseline readings taken immediately after adding LSMSNPs. After 24 h, L1:1 groups showed higher residual fluorescence intensity (2.50 ± 0.73) at inner Transwell compared with L1:0.5 (0.90 ± 0.45) and L1:0.25 (1.34 ± 0.67), suggesting that cell internalization of NPs also depends on different thickness of PLA coating.

Phorbol-myristate-acetate was then added in lower culture medium in order to stimulate the microglia to produce superoxide, which is expected to accelerate the degradation of PLA coating on LPMSNPs. After 24 h, all LPMSNPs showed higher amount of RSV release in the bottom compartment than the PMSNPs (particles without the LDL peptides), suggesting that LDL peptide effectively facilitated the transport of PMSNPs across the endothelial cell layer. It is also clear that without the PMA stimulation, the release of RSV is minimum, indicating the ROS responsiveness of the PMSNPs. Notably, there is a significant difference of RSV concentration among L1:1 group and L1:0.25 or L1:0.5 groups, with less release from thicker coatings (Fig. 7b). This indicates that the degradation of PLA and release rate of RSV is dependent on the thickness of PLA coating.

**Fig. 8:**
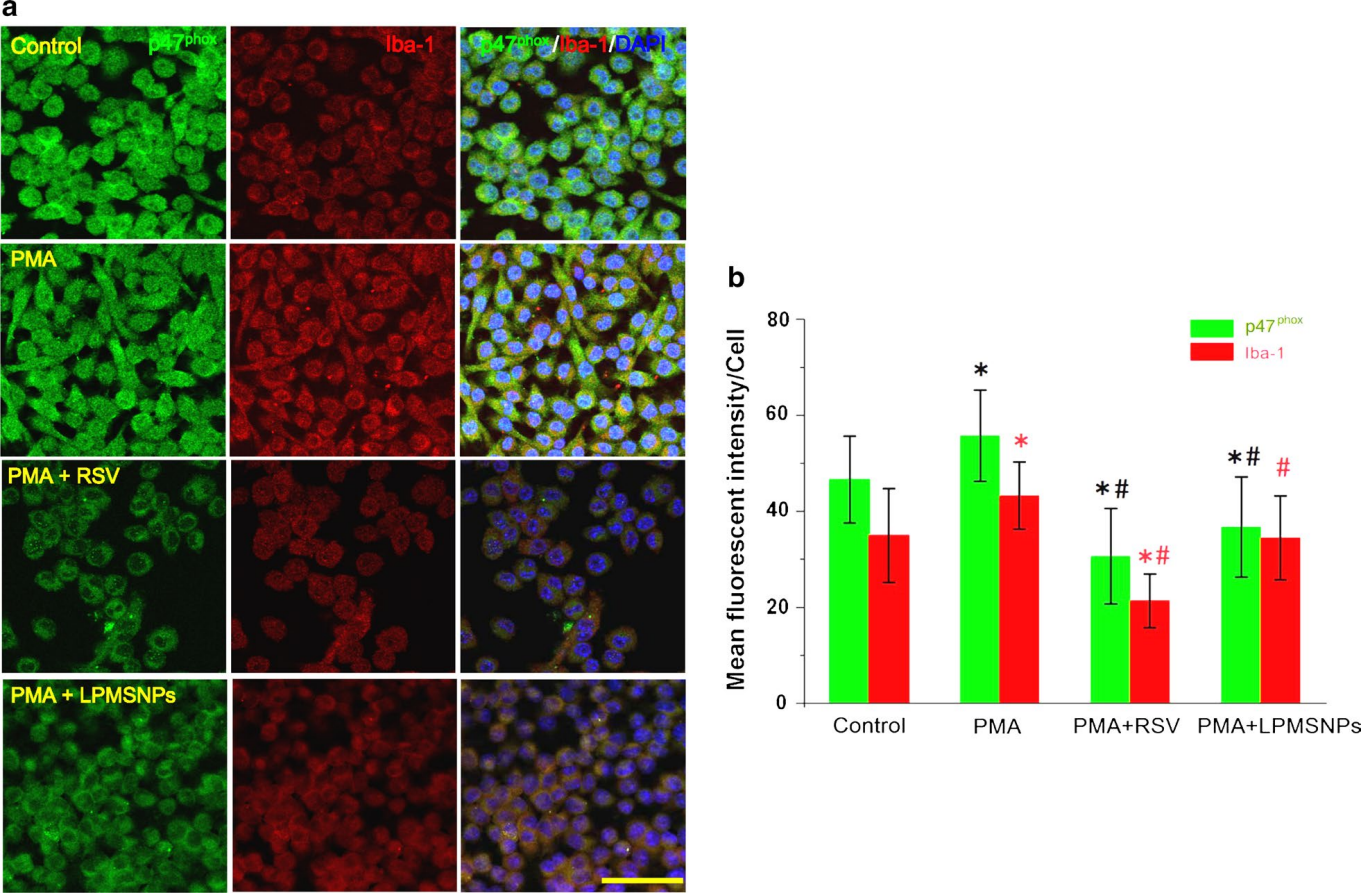
The effect of RSV released from LPMSNPs on PMA activated microglia. **a** The expression of NADPH p47^phox^ and Iba-1 by immunofluorescence assay. PMA activates NADPH p47^phox^ and up-regulates Iba-1 in HAPI microglia cells and increase superoxide production, which could be reduced by RSV. **b** The quantitive analysis of mean fluorescence intensity. *p < 0.05 vs control; ^#^*p* < 0.05 vs only PMA stimulation

**Fig. 9:**
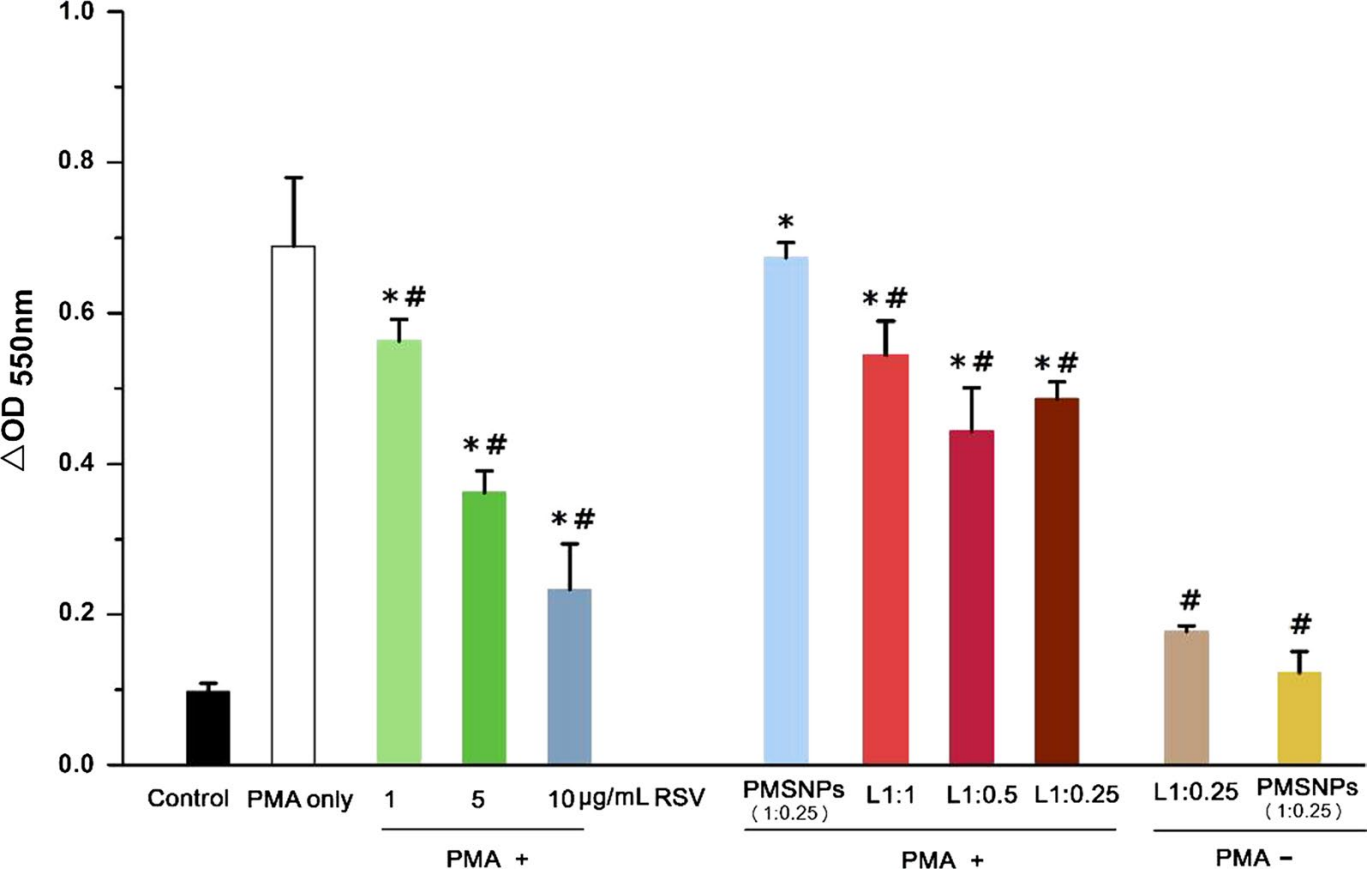
Quantification of the superoxide production in PMA stimulated microglia culture in the lower chamber and the effect of RSV releasing LPMSNPs. The superoxide is quantified by absorbance of reduced cytochrome c at 550 nm. *p < 0.05 vs control; ^#^*p* < 0.05 vs only LPS/IFNγ stimulation stimulation

Phorbol-myristate-acetate triggers superoxide production from microglia via inducing phosphorylation of NADPH oxidase subunit p47^phox^ and initiating activation of the neutrophil oxidase. Accordingly, the expressions of p47^phox^ and microglia activation marker Iba-1 were further investigated by double immunofluorescence staining and the quantification of fluorescence intensity was performed in ImageJ. As shown in Fig. 8a, in the control group, the HAPI cells showed typical resting morphology with small cell bodies. PMA significantly upregulated p47^phox^ and Iba-1 production in HAPI cells accompanied with a morphological change from polygon to elongated spindle. The addition of 10 μg/mL free RSV significantly suppressed the expressions of p47^phox^ and Iba-1 in PMA-stimulated HAPI cells, and recovered their original flat polygon morphology. As similar to direct addition of soluble RSV, it could be found that LPMSNPs (1:0.25) also induced morphologic changes (Fig. 8a) and significantly decreased expressions of p47^phox^ and Iba-1 in PMA-treated HAPI cells (Fig. 8b), indicating that RSV could release from LPMSNPs to inhibit activation of microglia. The result of cytochrome c assay showed that there was a very low ferrocytochrome c absorbance at 550 nm (ferricytochrome c reduction) in control group, while a highest absorbance could be found in PMA stimulation only group. Setting these two groups (control and PMA only groups) as references, we found that 1, 5 and 10 μg/mL free RSV could significantly reduce ferrocytochrome c absorbance. Importantly, there are significant differences of ferrocytochrome c absorbance among all of the LPMSNP groups (L1:1, L1:0.5, L1:025) after PMA stimulation and stimulated control groups (*p* < 0.05), as RSV released from NPs considerably inhibited superoxide production induced by PMA. Compared to free RSV addition, the ferrocytochrome c absorbance in L1:1 group almost equated to that in 1 μg/mL free RSV; whereas the absorbance in L1:0.5 and L1:025 groups was lower than that in 1 μg/mL but higher than that in 5 μg/mL free RSV group. Consistent with the results of Fig. 7b, all LPMSNPs showed lower OD than the PMSNPs, suggesting that little RSV was delivered to the microglia in the lower chamber via PMSNPs. This result confirmed that particles without the LDL peptides have low ability across the RBECs barrier. Additionally, both of L1:0.25 and PMSNPs groups in the absence of PMA stimulation (PMA−) showed lower absorbance compared to PMA only groups, suggesting non-stimulated microglia do not produce sufficient ROS to trigger PLA degradation and induce RSV release (Fig. 9).

**Fig. 10:**
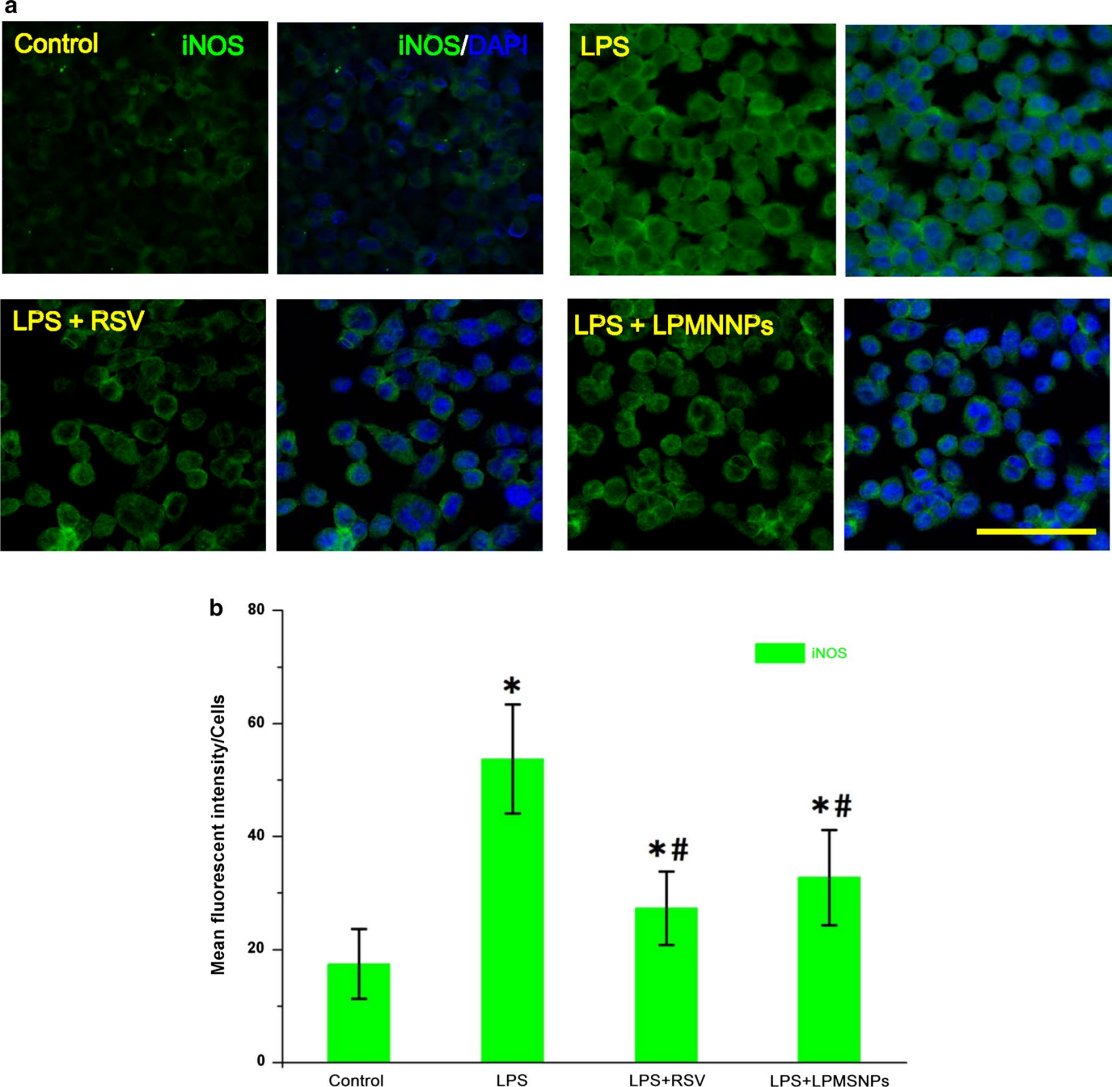
The effect of RSV released from LPMSNPs on LPS activated microglia via expression and distribution of iNOS on microglia. **a** The expression of iNOS by immunofluorescence assay. **b** The statistical analysis of mean fluorescence intensity. *p < 0.05 vs control; ^#^*p* < 0.05 vs only PMA stimulation

**Fig. 11:**
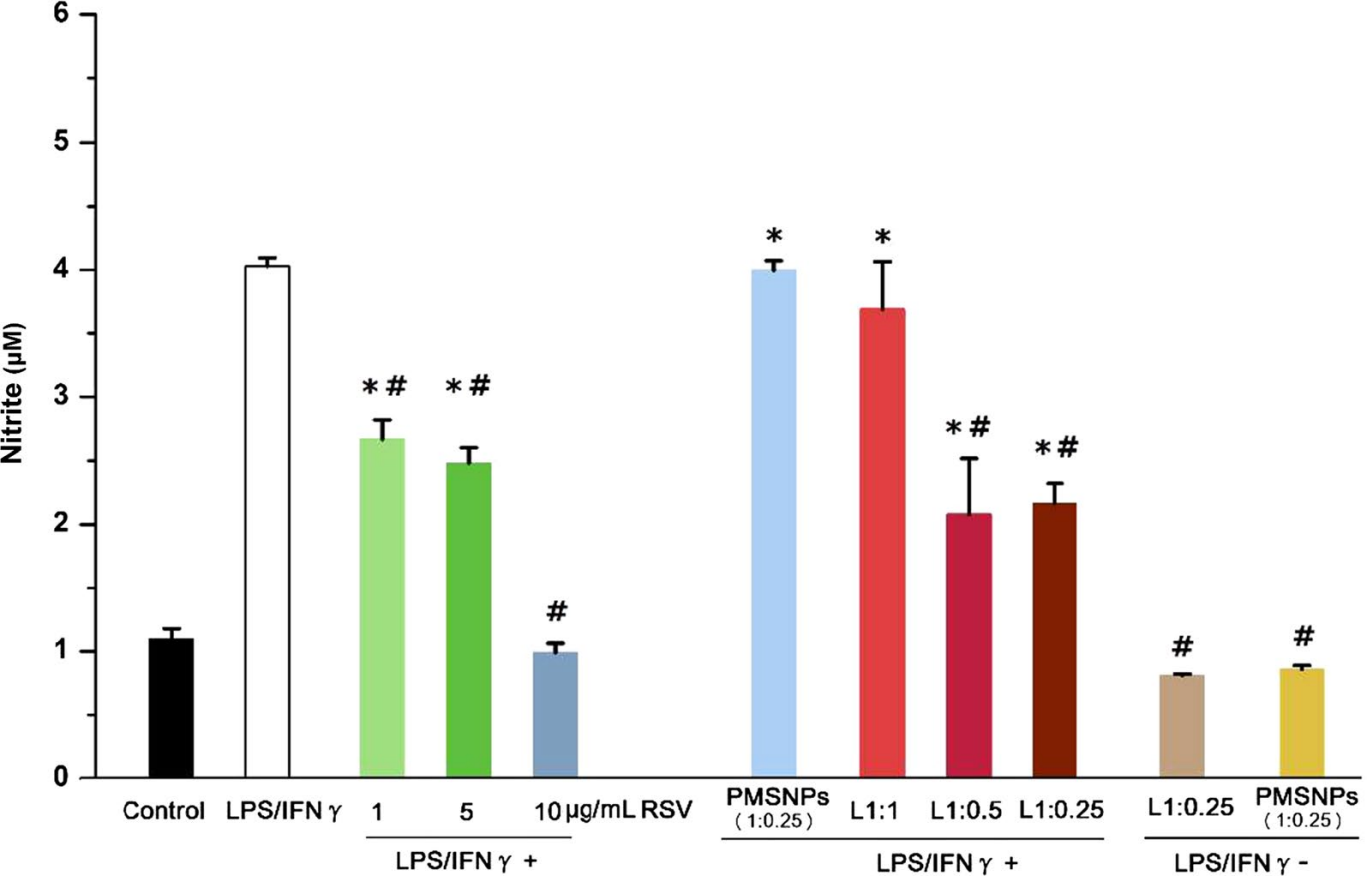
Quantification of the nitric production (nitrite) by Griess assay in LPS stimulated microglia and effect of RSV releasing LPMSNPs. *p < 0.05 vs control; ^#^*p* < 0.05 vs only LPS/IFNγ stimulation

Lipopolysaccharide is a potent immune system stimulant to induce microglial activation, and lead to release of proinflammatory cytokines and in turn activating inducible iNOS. Our results showed that exposure to LPS/IFNɣ markedly increased the expression of iNOS in HAPI cells, which could be significantly suppressed by free RSV or RSV-loaded in LPMSNPs (Fig. 10a, b). After exposure to LPS/IFNɣ for 24 h, there was a significantly increased nitrite production from HAPI cells. Treatment with free RSV (1, 5, 10 μg/mL) significantly inhibited LPS/IFNɣ-induced NO release (Fig. 11). Similar to the result of cytochrome c reduction assay, there were significant decreases in nitrite concentration among all LPMSNPs (L1:1, L1:0.5, L1:0.25) with LPS/IFNɣ stimulation compared to the LPS/IFNɣ stimulation only group. The nitrite concentration in L1:0.5 and L1:0.25 group shows significant differences compared to that in L1:1 group, suggesting that the thickness of PLA play a role in controlling RSV delivery. PMSNPs with LPS/IFNɣ did not reduce the nitrite production suggesting that without the peptide ligand, PMSNPs can not pass BBB to have a therapeutic effect to the microglia in the bottom well. Also, both L1:0.25 and PMSNPs groups in absence of LPS/IFNɣ stimulation (LPS/IFNɣ−) showed low NO production (Fig. 11). These results further confirmed that the LPMSNPs can pass the in vitro BBB and release RSV to effectively reduce the inflammation of microglia and the consequent production of superoxide and nitric oxide.

## Discussion

The BBB is a highly selective permeability barrier that separates the peripheral blood from CNS in order to maintain optimal levels of essential nutrients and neurotransmitters in the brain [9]. Together with the blood-cerebrospinal-fluid barrier, BBB plays a critical role in protecting the CNS against neurotoxins and regulating the homeostasis of the brain. However, it also limits the transport and delivery of biopharmaceutical drugs to the CNS. To overcome this challenge, we developed a new LPMSNPs-based drug delivery system with the goal of delivering antioxidants to combat oxidative stress in the brain tissue, which is thought to be associated with various neurological injuries and disorders.

For MSNPs, drug loading efficiency and dissolution rate are heavily dependent on pore morphology, pore size and loading method [12]. For the present study, commercial MSNPs with particle size 200 nm and pore size 4 nm were chosen. MSNPs with a pore size ranging from 2 to 50 nm have been reported to be excellent candidates for drug delivery and biomedical applications [19]. Although some studies suggest nanoparticles with smaller size are easier to cross BBB, plenty of literatures exist showing successful crossing of BBB in vivo with particle size similar to or larger than 200 nm [20–22]. Additionally, particles with sizes between 120 and 200 nm can escape the reticuloendothelial system allowing longer retention time in the circulation for RSV to bypass the liver and spleen filtration, which would result in increased BBB contact and crossing [23].

Here, we showed that 16 μg RSV can be loaded into 1 mg of MSNPs after 3 days of physical adsorption, and the MSNPs rapidly release 90% of drug load after 5 days. Driven by the need for on-demand drug delivery, ROS-responsive materials have gained increasing interests [24, 25]. To achieve the ROS responsive release of drug in a suitable concentration at the desired target area, various MSNPs-based stimuli-responsive drug release systems were developed using polyelectrolytes, lipid bilayer, or degradable polymers as gatekeepers [26–28]. In our study, PLA was chosen as a gatekeeping layer of MSNPs due to the ROS responsiveness of the polymer [29]. We found that PLA layer could significantly inhibit the release of the RSV from MSNPs in PBS, but was rapidly degraded by the presence of superoxide or the inflamed extracellular environment of activated microglia. With this mechanism, a true ROS responsive release system can be realized. Excess RONS produced in diseased or injured CNS can act as the demand stimulus to trigger the release of antioxidant compound to reduce oxidative stress. In this study, the TEM and SEM evidences demonstrated that PLA polymer can be successfully prepared on the MSNPs by solution blending method. The colloidal stability and dispersity of the fabricated nanoparticles is very important for the biological applications of nanoparticles [30]. The Zeta potential in PBS solution of PLA NPs or PLA-coated NPs are − 49 mV [31] and − 63.95 ± 1.29 mV [32], respectively, suggesting that both of PLA NPs and PLA-coated NPs for drug delivery system as stable drug carriers. In our dynamic light scattering (DLS) experiment, the PLA coated MSNPs suspensions were stable over the period of 1 h with no observable changes in the size distribution of the particles (Additional file 1: Figure S3).

By varying in the concentration of PLA in the initial coating solution, we can adjust the thickness of the coating on the MSNPs. There were no differences of fluorescence intensity of FTIC-labeled peptide among three groups (L1:1, L1:0.5, L1:0.25) (Fig. 7a), suggesting that thickness of PLA coating does not affect the amount of peptide binding. This is to be expected as the peptides are bounded only to the surface. On the other hand, RSV release in PBS containing superoxide (Fig. 3) or in Transwell chamber with stimulation of PMA and LPS (Fig. 7b) were dependent on the thickness of the PLA layer. Also, the results of reduced cytochrome c (Fig. 9) and nitrite concentration (Fig. 11) indicated significant difference among L1:1 group and L1:0.25 or L1:0.5 groups, suggesting that the thickness of PLA play a role in controlling RSV delivery. However, the well-controlled thickness of PLA coating and matched RSV release rate for curing superoxide will be examined in our future study. Additionally, the future experiments will also explore the effect of different structure and molecular weight of PLA on drug release profiles.

To enhance the recognition and internalization by RBECs, the surface of PLA-coated MSNPs was functionalized with LDL peptide receptor. Receptor-mediated transcytosis has been suggested as a promising noninvasive route to target and cross the BBB [33]. In this study, a novel LDLR ligand peptide (Ac-[cMPRLRGC]c-NH_2_) was used to enhance transcytosis. We have found that successful conjugation of LDLR ligand peptides by EDC/NHS chemistry markedly enhanced the migration of MSNPs across the RBECs monolayer. Both TEM and confocal microscopy confirmed the internalization of the LPMSNPs by RBECs, suggesting that this peptide is a promising tool to effectively facilitate the transport across BBB.

To test the efficiency of the RSV delivery across BBB and the efficacy in reducing RONS, an in vitro co-culture model was built. Previously in vitro BBB models have been built with various endothelial cells cultured on semiporous membranes of Transwell in the presence of pericytes, astrocytes, and/or neurons in different arrangements [34–36]. In our model, primary cerebral vascular endothelial cells were chosen to form the barrier. With this barrier model, we have demonstrated that the fluorescently labeled LPMSNPs crossed the BBB successfully. To assess the efficacy of the drug release, we added PMA or LPS stimulated microglia cells to the co-culture system to mimic inflammatory environment. It is well established that PMA increases •O_2_^−^ production via protein kinase C (PKC)-induced activation of the NADPH oxidase subunit p47^phox^ from activated microglia with the elevation of intracellular calcium [37]. The stimulation of the NADPH oxidase in PMA-activated rat microglia induces peroxynitrite production [38]. LPS activates microglia to produce ROS and RNS through p53 and iNOS mediated pathway [39]. Comparing primary microglia and immortalized cells lines (BV-2 and HAPI cells), Horvath et al. [40] indicated that LPS treatment induced NO release from all cells cultures at 24 h. Based on this result, we chose to use the HAPI cell line for its ease of use and consistency.

As an important antioxidant against oxidative stress, RSV has not been used clinically due to its low bioavailability in specific desired locations including CNS [41]. Nanoencapsulation of RSV is likely to provide protection against degradation, enhancement of bioavailability, improvement in intracellular penetration and more controlled release profile [42, 43]. Using liposomes for RSV loading, Kristl et al. [44] indicated that 10 μM RSV induced a change in metabolic activity of HEK293 cells and significantly improved antioxidative capacity. Frozza et al. [45] demonstrated that RSV-loaded lipid-core nanocapsules increased the concentration of RSV in the rat brain tissue. Additionally, RSV-loaded solid lipid NPs (< 200 nm) functionalized with apolipoprotein E showed a significant increase through hCMEC/D3 monolayers compared to non-functionalized NPs [46]. In this study, RSV was used as a model drug to alleviate effects of oxidative stress in activated microglia. Our results indicated that the release of RSV from MSNPs could effectively reduce the inflammation of activated microglia and the consequent production of superoxide and nitric oxide. Combined with the targeting role of LDLR ligand peptides and ROS responsiveness of the PLA gate keeper, the LPMSNPs showed great potential for ROS responsive delivering RSV to eliminate oxidative stress in CNS.

We determined that the highest loading content of RSV into 1 mg MSNPs is about 16 μg (Additional file 1: Figure S2) and the release of RSV in PBS after stimulation with XO and PR was negligible after 48 h. On the other hand, RSV release from PMSNP in the co-culture after PMA stimulation reached ~ 0.9–1.4 μg for various LPMSNPs (Fig. 7b). The faster release suggests that the activated microglia might have generated more reactive species that could accelerate the PLA degradation. Nevertheless, with the total drug load of 16 μg, a sustained release over many days can be expected.

## Conclusions

In this study, we developed an LDLR ligand peptide conjugated with different mass ratio of PLA-coated MSNPs for RSV delivery targeting oxidative stress in CNS. Using an in vitro model of BBB/inflammation, our results confirmed that LDLR peptides enhance the transcytosis of MSNPs and the crossing of BBB, the RSV could be released from PLA-coated MSNPs in response to artificially added superoxide or RONS released by PMA or LPS activated microglia, and the released RSV reduce oxidative stress. Taken together, our RSV delivery system showed great potential for treating various CNS disorders associated with oxidative stress. Future work will be conducted in vivo to demonstrate the therapeutic efficacy of the RSV delivery.

## Additional file

**Additional file 1: Figure S1.** Characterization of eluted PLA from MSNPs by NMR. The NMR spectra of PLA (A) and coated PLA with 1:0.25 (B), 1:0.5 (C) and 1:1 (D) mass ratio on MSNPs. PLA coated MNSPs at different ratios were re-dissolved in CDCl_3_ solvent and the solutions were analyzed by NMR. **Figure S2.** RSV adsorption profile onto MSNPs from 30 μg/mL RSV solutions. The RSV was dissolved in 50% ethanol/PBS solution and then mixed with MSNPs at room temperature. The mixture was centrifuged at specified time point, and the supernatant was analyzed with spectroscopy at 304 nm to determine the amount of free RSV remaining in solution and the amount of RSV loaded in the MSNPs was determined by subtracting the remaining RSV from the total amount originally present. n = 3. **Figure S3.** Dynamic Light Scattering measurement displaying the hydrodynamic diameters for uncoated mesoporous silica nanoparticles and PMSNPs in water over 1 h. DLS measurements were performed by Malvern ZS90 Zetasizer. 1:0.25 PMSNPs were suspended at 0.5 mg/ml in water then pipetted into a disposable polystyrene cuvette for analysis. Measurements were taken every 15 min. **Table S1.** Sample identification codes and preparation conditions.

## Abbreviations

BBB: blood–brain barrier
CNS: central nervous system
EDC: *N*-(3-dimethylaminopropyl)-*N*′-ethylcarbo-diimide
IFNɣ: interfeuron gamma
LDLR: low-density lipoprotein receptor
LPMSNPs: FITC-labeled LDL peptides conjugated with PMSNPs
LPS: lipopolysaccharide
MSNPs: mesoporous silica nanoparticles
NHS: *N*-hydroxysuccinimide
NPs: nanoparticles
PLA: polylactic acid
PMA: phorbol-myristate-acetate
PMSNPs: PLA-coated MSNPs
PR: 2-amino-4-hydroxypteridine Pterine
RBECs: brain microvascular endothelial cells
RNS: reactive nitrogen species
RONS: reactive oxygen and nitrogen species
ROS: reactive oxygen species
RSV: Resveratrol
SOD: superoxide dismutase
TEER: transendothelial electrical resistance
XO: Xanthine oxidase
PKC: protein kinase C
