# Supplementary material for: ROS responsive resveratrol delivery from LDLR peptide conjugated PLA-coated mesoporous silica nanoparticles across the blood–brain barrier
Source: J Nanobiotechnology. 2018 Feb 13;16:13. doi: 10.1186/s12951-018-0340-7 (PMC5810018; doi:10.1186/s12951-018-0340-7)
Supplement: Supplementary file 1 — Additional file 1: Figure S1. Characterization of eluted PLA from MSNPs by NMR. The NMR spectra of PLA (A) and coated PLA with 1:0.25 (B), 1:0.5 (C) and 1:1 (D) mass ratio on MSNPs. PLA coated MNSPs at different ratios were re-dissolved in CDCl3 solvent and the solutions were analyzed by NMR. Figure S2. RSV adsorption profile onto MSNPs from 30 μg/mL RSV solutions. The RSV was dissolved in 50% ethanol/PBS solution and then mixed with MSNPs at room temperature. The mixture was centrifuged at specified time point, and the supernatant was analyzed with spectroscopy at 304 nm to determine the amount of free RSV remaining in solution and the amount of RSV loaded in the MSNPs was determined by subtracting the remaining RSV from the total amount originally present. n = 3. Figure S3. Dynamic Light Scattering measurement displaying the hydrodynamic diameters for uncoated mesoporous silica nanoparticles and PMSNPs in water over 1 h. DLS measurements were performed by Malvern ZS90 Zetasizer. 1:0.25 PMSNPs were suspended at 0.5 mg/ml in water then pipetted into a disposable polystyrene cuvette for analysis. Measurements were taken every 15 min. Table S1. Sample identification codes and preparation conditions. [file 12951_2018_340_MOESM1_ESM.docx]

**Additional Information**

**ROS Responsive** **Resveratrol Delivery from LDLR Peptide Conjugated PLA-coated Mesoporous Silica Nanoparticles Across the Blood-Brain Barrier**

Yang Shen ^a,b^, Bin Cao ^a^, Noah R. Snyder ^a^, Kevin M. Woeppel^a,c^, James R. Eles ^a^, Xinyan Tracy Cui ^a,c,d^ *

*^a^ Department of Bioengineering, University of Pittsburgh, Pittsburgh, PA 15260, USA*

*^b^ Institute of Biomedical Engineering, School of Preclinical and Forensic Medicine, Sichuan University, Chengdu 610041, China*

*^c^Center for the Neural Basis of Cognition, University of Pittsburgh, Pittsburgh, PA, USA 
^d^McGowan Institute for Regenerative Medicine, University of Pittsburgh, Pittsburgh, PA 15260, USA*

*^*^Corresponding author, 5057 Biomedical Science Tower 3, 3501 Fifth Avenue, Pittsburgh, PA, USA. E-mail:*[*xic11@pitt.edu*](mailto:xic11@pitt.edu)*; Fax: +1-412-648-9076; Tel: +1-412-383-6672*


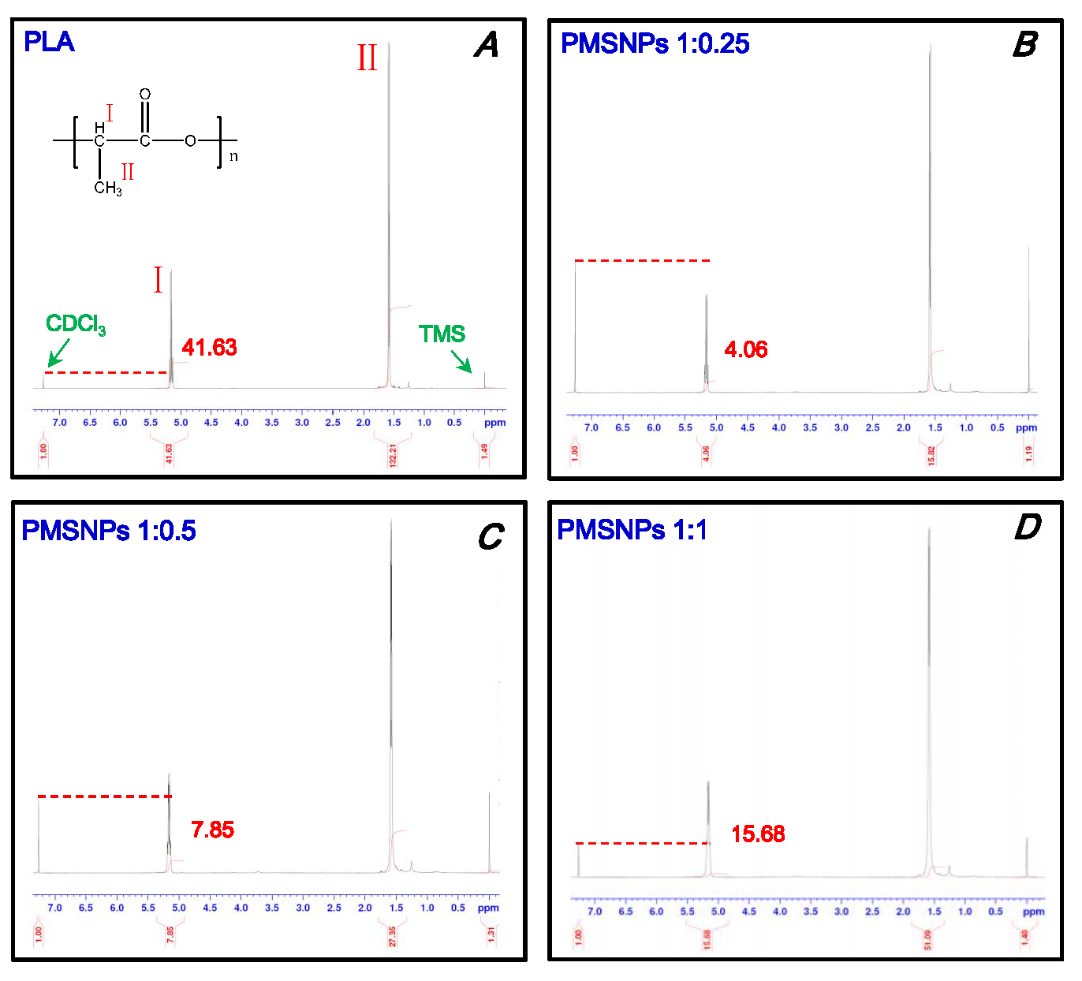


**Figure S1.** Characterization of eluted PLA from MSNPs by NMR. The NMR spectra of PLA (A) and coated PLA with 1:0.25 (B), 1:0.5 (C) and 1:1 (D) mass ratio on MSNPs. PLA coated MNSPs at different ratios were re-dissolved in CDCl_3_ solvent and the solutions were analyzed by NMR.


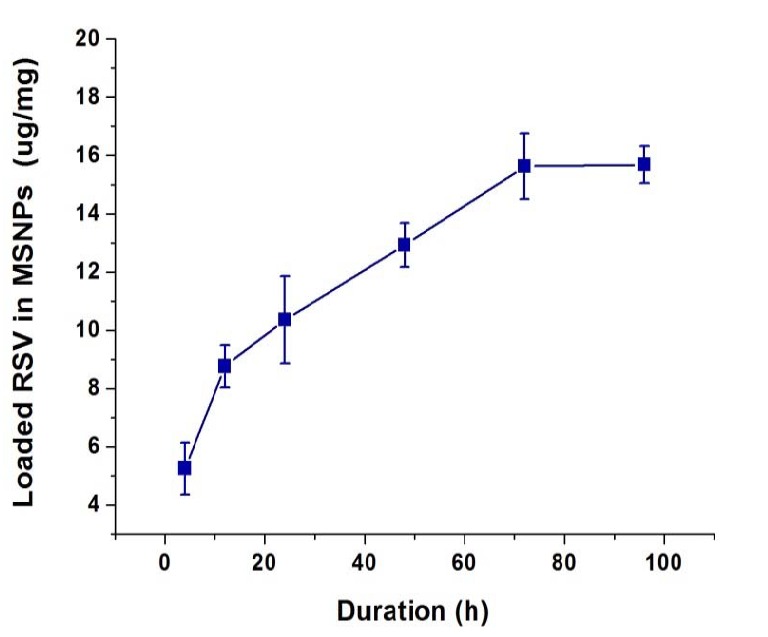


**Figure S2.** RSV adsorption profile onto MSNPs from 30μg/mL RSV solutions. The RSV was dissolved in 50% ethanol/PBS solution and then mixed with MSNPs at room temperature. The mixture was centrifuged at specified time point, and the supernatant was analyzed with spectroscopy at 304 nm to determine the amount of free RSV remaining in solution and the amount of RSV loaded in the MSNPs was determined by subtracting the remaining RSV from the total amount originally present. n=3.


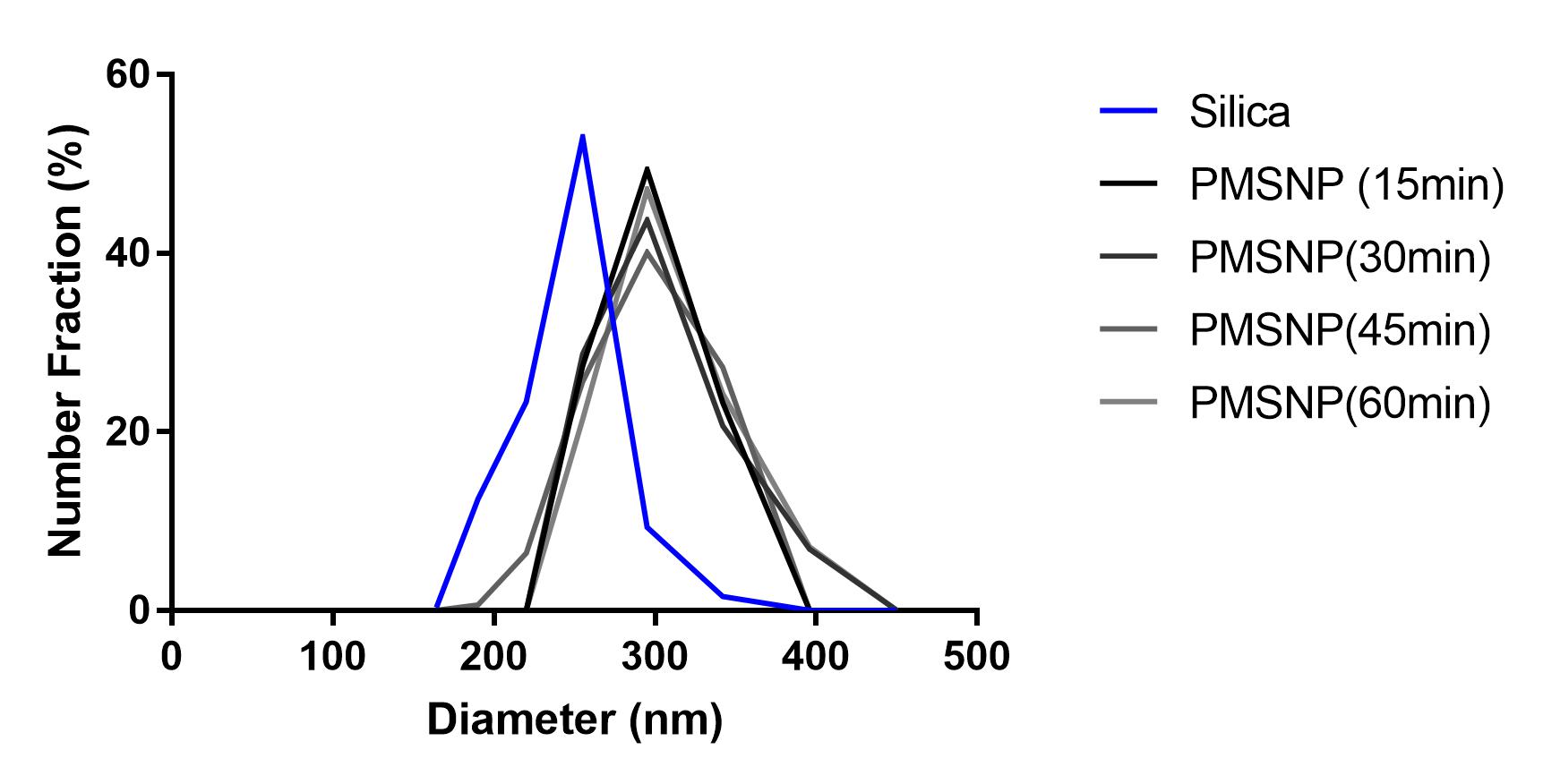


**Figure S3**: Dynamic Light Scattering measurement displaying the hydrodynamic diameters for uncoated mesoporous silica nanoparticles and PMSNPs in water over 1 hour. DLS measurements were performed by Malvern ZS90 Zetasizer. 1:0.25 PMSNPs were suspended at 0.5mg/ml in water then pipetted into a disposable polystyrene cuvette for analysis. Measurements were taken every 15 mins.

**Table S1. Sample identification codes and preparation conditions**

| **Identification Codes** | **Drug loading** | **PLA coating** | **FITC-labeled LDL peptide binding** |
| --- | --- | --- | --- |
| **MSNPs (mesoporous silica nanoparticles)** | | | |
| **1:0** | 30μg/mL RSV in 10mg MSNPs | - | - |
| **PMSNPs (PLA-coated mesoporous silica nanoparticles)** | | | |
| **1:1** | 30μg/mL RSV in 10mg MSNPs | PLA: MSNPs =10mg:10mg | - |
| **1:0.5** | 30μg/mL RSV in 10mg MSNPs | PLA: MSNPs =5mg:10mg | - |
| **1:0.25** | 30μg/mL RSV in 10mg MSNPs | PLA: MSNPs =2.5mg:10mg | - |
| **LPMSNPs (****FITC-labeled LDL peptides conjugated with PLA-coated MSNPs)** | | | |
| **L1:1** | 30μg/mL RSV in 10mg MSNPs | PLA: MSNPs =10mg:10mg | 10μg/mL peptide, 4h |
| **L1:0.5** | 30μg/mL RSV in 10mg MSNPs | PLA: MSNPs =5mg:10mg | 10μg/mL peptide, 4h |
| **L1:0.25** | 30μg/mL RSV in 10mg MSNPs | PLA: MSNPs =2.5mg:10mg | 10μg/mL peptide, 4h |
